# Optimizing yield and water productivity in summer mung bean (*Vigna radiata* L.) through crop residue management and irrigation strategies

**DOI:** 10.1186/s12870-024-05640-1

**Published:** 2024-10-28

**Authors:** Saurabh Tripathi, Anureet Kaur, Ajmer Singh Brar, Karamjit Singh Sekhon, Sukhpreet Singh, Anurag Malik, Ozgur Kisi

**Affiliations:** 1 Department of Agronomy, Punjab Agricultural University, Ludhiana, Punjab India; 2 Punjab Agricultural University, Regional Research Station, Bathinda, 151001 Punjab India; 3 Department of Civil Engineering, University of Applied Sciences, 23562 Lübeck, Germany; 4 Department of Civil Engineering, Ilia State University, Tbilisi, 0162 Georgia; 5 School of Civil, Environmental and Architectural Engineering, Korea University, Seoul, 02841 South Korea

**Keywords:** Summer mung bean, Crop residue management, Irrigation regimes, Grain yield, Water productivity

## Abstract

A multi-season research trial entitled ‘crop residue management effects on yield and water productivity of summer mung bean (*Vigna radiata* L.) under different irrigation regimes in Indian Punjab’ was conducted at Punjab Agricultural University (PAU), Regional Research Station (RRS), Bathinda, during *rabi* 2020 and 2021. The field experiment was conducted in a split-plot layout with nine treatment combinations and replicated thrice. The treatments consisted of T_1_ (no wheat residue along with tillage), T_2_ (leftover wheat residue with zero tillage), and T_3_ (incorporated wheat residue along with tillage) in main plots and irrigation regimes viz., I_1_ (vegetative growth and flowering stage), I_2_ (vegetative growth, flowering, and pod filling stage) and I_3_ (vegetative growth, flowering, pod formation and pod filling stage) in sub-plots, respectively. The growth and yield attributing characters were significantly higher under T_3_ than T_1_ but statistically at par with T_2_ during both years. An increase of 24.1% and 19.0% in grain yield was found in residue incorporation (T_3_) and residue retention (T_2_) over residue removal (T_1_), respectively. Maximum crop and irrigation water productivity was observed under T_3_ due to reduced water use and increased yield. Among the irrigation regimes, the I_3_ recorded significantly higher grain yield (0.70 and 0.79 t ha^− 1^) than I_1_. It was at par with I_2_ during both years due to higher irrigation frequency at the pod formation and pod filling stage. Crop water productivity (CWP) was higher under I_3_, whereas irrigation water productivity (IWP) was higher under I_1_ during both years. Additional irrigation at the pod-filling stage increased the grain yield by 36.5%, and two additional irrigations at the pod-formation and pod-filling stage further increased yield by 46.2% compared to only two irrigations at the vegetative and flowering stages. The treatment combinations of T_2_I_2_ and T_3_I_2_ outperformed T_1_I_3_ in terms of growth and yield attributing characters viz. plant height, dry matter accumulation (DMA), leaf area index (LAI), pods plant^− 1^, seeds pod^− 1^, and 1000-seed weight, which resulted in higher grain yield in these treatment combinations over T_1_I_3_. Applying crop residue can help minimize water use and increase crop water productivity. So, retaining crop residue in summer mung bean resulted in saving irrigation water due to lesser evapotranspiration from the soil surface.

## Introduction

With the burgeoning global population and heavy reliance on agriculture for food security, there is a dire need to enhance agricultural production to feed the human population. India is a densely populated country with a significant under-nourished population, particularly children and women. A substantial proportion of children are suffering from protein malnutrition in the country [1] because most Indian people are vegetarian due to their religious beliefs. Under such a situation, consuming legumes or pulses, also known as “poor man’s meat,” regularly can help overcome protein malnutrition and provide an economical source of protein [2]. In Punjab, where the mono-culture of rice-wheat cropping system is widely adopted by farmers, a short-duration summer moong crop can fit well and serve as an additional source of income while providing a protein-rich diet to the rural population. This pulses-based cropping system, i.e., rice-wheat-summer moong, has been supported as an option in contrast to the persistent rice-wheat cropping system because of its ability to rejuvenate soil fertility by fixing environmental nitrogen. Including green gram in the rice-wheat cropping system also helps lower the fertilizer consumption in the case of rice crops particularly nitrogen, due to symbiotic nitrogen fixation by the green gram. Hence, green gram beans are the best option for the small window between harvesting wheat and transplanting rice.

Mung bean, popularly known as green gram, is an established and old legume crop with the family *Papilionoideae* and the primary center of origin in Southeast Asia [3]. It covers an area of about 4.5 M ha, with a production of 2.5 million tons and productivity of 548 kg ha^-1^ in India [4]. The crop has better nutritive worth and is affordable for consumers [3]. In terms of the nutrient composition of the mung bean seed, it consists of protein (20–24%), oil (2.1%), fats (2.05%), fiber (6.4%), moisture (9.4%), enough amount of Vitamin A and B, carbohydrates and 343.5 kcal energy per 100 g of seed [5]. Moreover, mung bean’s protein and carbohydrates are more quickly digestible than other legumes.

The state of Punjab is facing serious challenges in agriculture concerning declining groundwater, deteriorating soil health, crop residue management, etc. The groundwater resources of Punjab are on the verge of extinction due to the excessive withdrawal of this precious natural resource to raise rice crops. As per the latest report of Central Ground Water Board, out of 150 blocks in the state, 117 falls under the dark category, where groundwater withdrawal is way higher than the recharge. With maximum productivity and contribution of rice and wheat to the central food reserves, the state also produces vast amounts of rice and wheat crop residues. Due to the intensive cropping system, the farmers of the state resort to burning this residue as the most practical and economical way to dispose of agricultural waste [6] which results in severe air pollution and loss of precious nutrients from the system. Being a rich source of essential plant nutrients, this residue, if retained or incorporated in the soil, can serve as a multipurpose organic asset and revive the soil health [7–9]. The summer moong crop is grown from April to the end of June in Punjab between wheat harvest and rice transplanting and these are the months when the evaporative demand of the atmosphere is at its peak due to very high air temperatures and low relative humidity with hot winds. The retention or incorporation of wheat residue in summer moong crops not only enhances soil fertility and quality parameters [10, 11] but also helps reduce the loss of moisture as evaporation from the soil surface due to the mulching effect [12, 13]. Long-term retention and incorporation of crop residue results in lowering soil bulk density, enhancing the water retention capacity of the soil, increasing total soil porosity, improving soil water utilization efficiency [11, 14], and enhancing the soil’s enzymatic levels along with soil organic matter content [15]. This practice can, therefore, improve the soil’s physio-chemical and biological properties and crop water productivity.

The increasing danger of freshwater scarcity, continuous diminishing of arable land due to alternate usage, and frequently occurring droughts due to climate change have accelerated research on water-constrained irrigation techniques that aim to increase “crop per drop” production [16]. The optimum availability of soil moisture to the plant supplements the growth and dry matter production which ultimately increases the plant’s nutrient availability [17]. This can be achieved through efficient irrigation scheduling, which is vital for yield efficiency. Efficient irrigation scheduling aims to ensure the optimum availability of moisture to plants at critical growth stages without any wastage of irrigation water. Under limited water availability or in areas where there is a dire need to save groundwater resources, irrigation scheduling based on the critical growth stage of the crop synchronized with plant water requirements can play an important role in saving irrigation water without any yield penalty [18], It ultimately can improve the water-use efficiency of any crop [19]. In this context, deficit irrigation is a practicable strategy for saving water and enhancing water use efficiency with acceptable yields per water unit [20–22]. However, reducing irrigation water amounts more than normal could alter the physio-biochemical of plants [23–25]. Further, deficit irrigation resulted in weakness of membrane stability and stomata closure [26, 27], while influencing the nutrient uptake and utilization in plants [28, 29].

Keeping in mind the importance of crop residue management practices and efficient irrigation scheduling under Punjab conditions, a field experiment entitled “Crop residue management effects on yield and water productivity of summer mung bean under different irrigation regimes in Indian Punjab” was conducted at PAU, Regional Research Station, Bathinda during summer season of 2020 and 2021 with objectives (i) to study the effect of irrigation regimes and residue management practices on water productivity of summer mung bean, and (ii) to evaluate the interactive effect of irrigation regimes and residue management practices on water productivity of summer mung bean.

## Materials and methods

### Experimental site and weather condition

Field studies were carried out at the PAU, RRS, Bathinda (Punjab) during the summers of 2020 and 2021. Bathinda is located at latitude 30^o^ 36’ 09” N and a longitude 74^o^ 28’ 55” E, and an elevation of 211 m above sea level. The area’s climate is subtropical and semi-arid, characterized by three distinct seasons: hot, dry summers (April to June), hot, humid monsoons (July to September), and cold winters (November to January). Due to the subtropical climate, the average maximum temperature varies widely. In May and June, it often reaches up to 47 °C. However, in December and January, it’s not uncommon to experience freezing temperatures, frost, and cold winds, with night time lows of 0°C. The area receives an average annual rainfall of 440 mm, predominantly during the monsoon season from July to September.

**Fig. 1:**
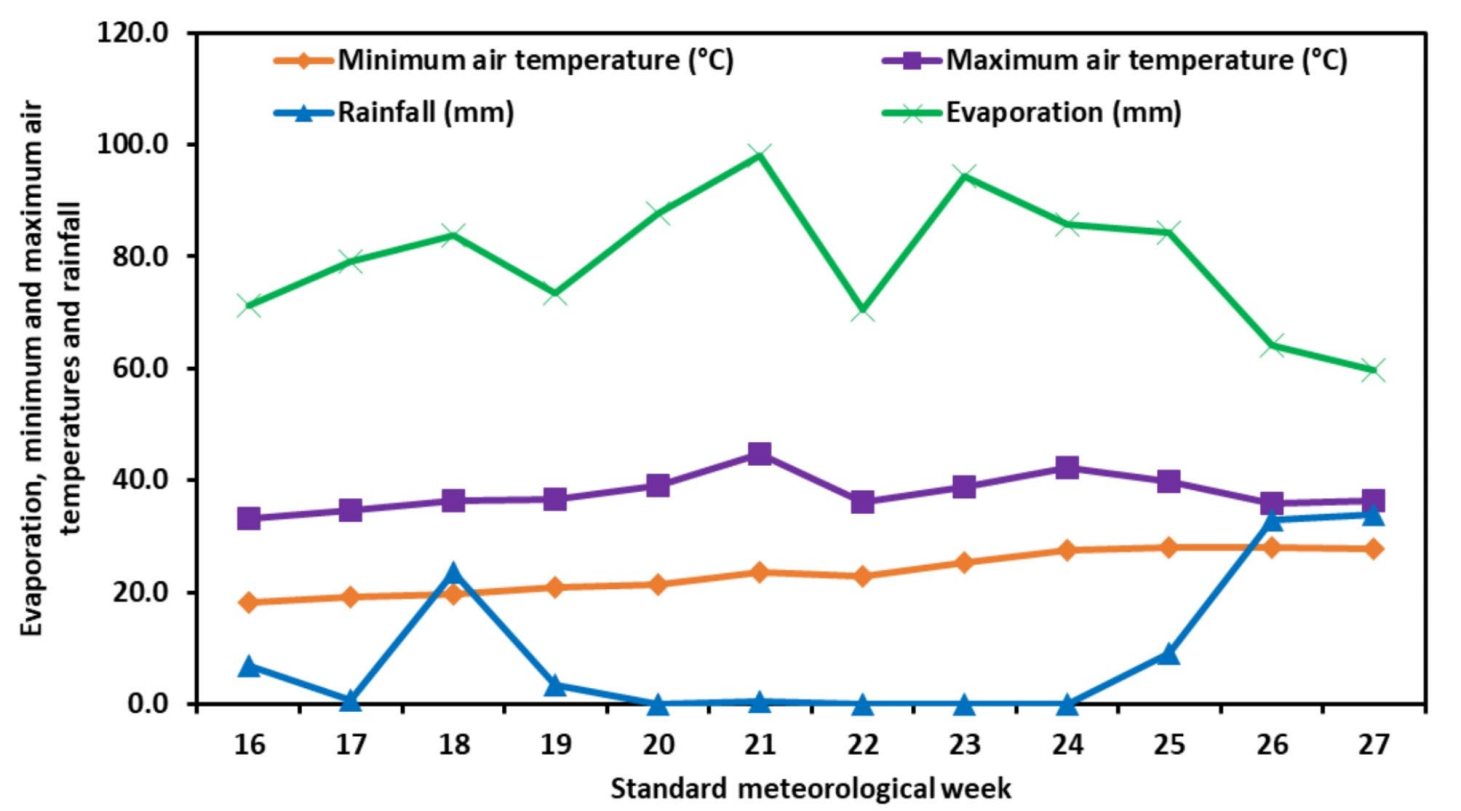
Weekly maximum air temperature (°C), minimum air temperature (°C), rainfall (mm) and evaporation (mm) during 2020

**Fig. 2:**
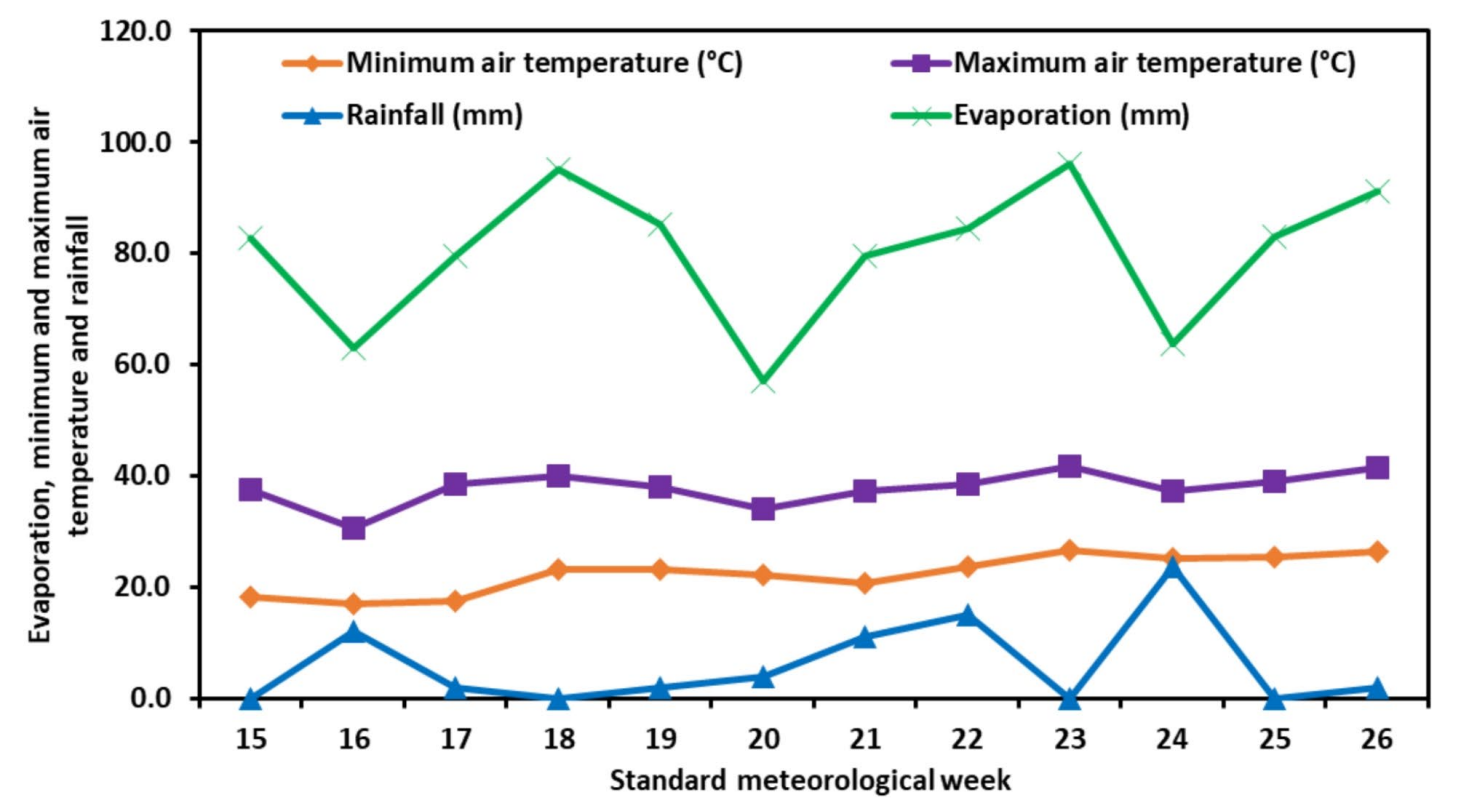
Weekly maximum air temperature (°C), minimum air temperature (°C), rainfall (mm) and evaporation (mm) during 2021

Meteorological data (as shown in Figs. 1 and 2) were collected from the meteorological observatory of PAU, RRS, Bathinda, during the crop growing seasons of 2020 and 2021, which span from the 15th to the 26th standard meteorological week (SMW) (April -July 2020 and 2021). During the summer months of 2020, the average weekly maximum air temperature ranged from 33.1 °C to 44.7 °C, while the average weekly minimum temperature varied between 16.2 °C and 28.0 °C. In the following year, 2021, the average weekly maximum temperature fluctuated between 30.5 °C and 41.7 °C, and the average weekly minimum temperature ranged from 17.1 °C to 26.7 °C. The average relative humidity during crop growth varied between 36.0 and 67.0% during 2020 and 37.9 to 72.0% during 2021. During 2020, the duration of sunshine in each week varied between 5.9 to 10.9 h, and in 2021, it ranged from 3.7 to 9.9 h. The growing season (16–27 SMW) saw a total rainfall of 111.4 mm and 72 mm, and the total evaporation measured was 952 mm and 960 mm for the years 2020 and 2021, respectively.

### Soil of the experimental field

The soil samples were collected from the experimental field before the start of the experiment from 0 to 100 cm depth (for physical properties) and 0–15 and 15–30 cm soil profiles (for chemical analysis) and were analyzed for initial soil physical and chemical properties. The data about these parameters is presented in Tables 1 and 2. The moisture content in the 0–100 cm soil profile at field capacity and permanent wilting point [30] was 26.5 and 12.2 cm, respectively, with an average soil bulk density of 1.59 g cm^-3^ [31]. The soil moisture in the 0–100 cm soil profile was 14.3 cm. In the 0–15 cm soil profile, the soil was sandy loam in texture with pH of 8.3 (1:2, soil: water suspension) measured by Beckman’s glass electrode pH meter [32] and an electrical conductivity (1:2, soil: water suspension) of 0.26 dS m^-1^ estimated by soil bridge conductivity meter [32]. The soil of the experimental field was low in available nitrogen (140.8 kg ha^-1^) and organic carbon content (0.23%), medium in available phosphorus (17.6 kg ha^-1^), and medium in available potassium (251.2 kg ha^-1^). The corresponding values for pH, EC, OC, available N, P, and K were 8.1, 0.21 dS m^-1^, 0.22%, 146.3 kg ha^-1^ (low), 16.81 kg ha^-1^ (medium) and 256.2 kg ha^-1^ (medium), respectively in 15–30 cm soil profile.

**Table 1:** Physical properties of soil profile of the experimental field

Soil depth (cm)	Field capacity moisture (cm)	Permanent wilting point moisture (cm)	Bulk density (g cm^− 3^)
0–10	3.08	0.86	1.57
10–20	3.39	0.98	1.61
20–30	3.61	1.89	1.60
30–40	4.93	2.41	1.57
40–60	5.25	2.96	1.61
60–100	6.24	3.10	1.59
Total	26.50	12.20	Average = 1.59

**Table 2:** Chemical characteristics of the soil

Chemical properties	Soil depth (cm)	
	0–15	15–30
pH (1:2, soil: water suspension)	8.3	8.1
Electrical conductivity (dS m^− 1^)	0.26	0.21
Organic carbon (%)	0.23	0.22
Available N (kg ha^− 1^)	140.8	146.3
Available P (kg ha^− 1^)	17.87	16.81
Available K (kg ha^− 1^)	251.2	256.2

### Experimental treatments and methodology

The experiment was conducted in a split-plot design with three crop residue management treatments. T_1_: Removal of wheat residue followed by preparatory tillage (No wheat residue along with tillage); T_2_: Zero tillage in the left-over wheat residue after making wheat straw with a straw reaper (left-over wheat residue with zero tillage) and T_3_: Incorporation of left-over wheat residue after making wheat straw with a straw reaper (Incorporation of left-over wheat residue with tillage) in the main plots and three irrigation regimes viz. I_1_: irrigation at the vegetative growth and flowering stage; I_2_: irrigation at the vegetative growth, flowering, and pod-filling stage; and I_3_: irrigation at the vegetative growth, flowering, pod-formation, and pod-filling stage in the sub-plots replicated thrice. The wheat residue was analyzed and contained 42.1% carbon, 0.47% N, 0.06% P, and 1.12% K with a C: N ratio of 89.6.

A short-duration spring/summer mung bean variety SML 668 was selected for research study as it can fit well in a rice-wheat-summer mung bean cropping system. The variety SML 668 was developed by Punjab Agricultural University (PAU), Ludhiana (Punjab), released and notified (Seed net Gazette Notification number 283, dated 12-03-2003) for Punjab state during 2003. In addition to Punjab, it became popular and covered large areas in Haryana, Rajasthan, Bihar, and Himachal Pradesh. The seed was acquired for research trial from Director Seeds, PAU, Ludhiana. The sowing of summer moong was done on 19th April 2020 and 12th April 2021, and harvesting on 7th July and 26th June 2020 and 2021, respectively. The crop was sown at a row-to-row spacing of 22.5 cm while plant-to-plant spacing was maintained at 7.0 cm. The seed was treated with Captan @ 3 g kg^-1^ before planting to keep the crop disease-free. The inoculation was done with a consortium biofertilizer @ 500 g for 15 kg seed containing *Rhizobium* sp. (LSMR-1) and Rhizobacteria (RB-3) mixed with water followed by drying in the shade. The recommended dose of nitrogen (12.5 kg ha^-1^) and P_2_O_5_ (40 kg ha^-1^) was applied to the crop at sowing in the form of urea and single superphosphate (SSP). The weed infestation was checked through hand-weeding after four weeks of sowing and hoeing at 45 days after sowing. A uniform pre-sowing irrigation of 75 mm was given to all the experimental plots, whereas post-sowing irrigations were applied as per the irrigation regimes. The details of the treatments are given in Table 3.

**Table 3:** Details of the treatments

Treatments	Symbols
Main Plot (wheat residue management)	
1. Preparatory tillage after removal of wheat residue (No wheat residue along with tillage)	T_1_
2. Zero tillage in the wheat residue left over after making wheat straw by a straw reaper (Leftover wheat residue with zero tillage)	T_2_
3. Incorporation of left-over wheat residue after making wheat straw along with preparatory tillage (Incorporated wheat residue along with tillage)	T_3_
Sub-plots (Irrigation levels)	
1. Two irrigations (I_1_: Vegetative growth and flowering stage)	I_1_
2. Three irrigations (I_2_: Vegetative, flowering and pod filling stage)	I_2_
3. Four irrigations (I_3_: Vegetative, flowering, pod formation and pod filling stage)	I_3_

During 2020, the I_1_ was scheduled on 6th May and 20th May, the I_2_ was scheduled on 6th May, 20th May, and 11th June, and the I_3_ was scheduled on 6th May, 20th May, 29th May, and 11th June respectively. Whereas during 2021, the I_1_ was scheduled on 14th May and 28th May, the I_2_ was scheduled on 14th May, 28th May, and 25th June, and the I_3_ was planned on 14th May, 28th May, 11th June, and 25th June, respectively. The preparatory tillage was given in the main plots based on the treatments. The crop was sprayed with Quinalphos @ 2000 ml ha^-1^ to save the crop from insect pest damage. The harvesting was carried out when 80% of the pods showed maturity. The net plot size left for harvesting was 6.0 × 3.15 m (18.9 m^2^) after leaving one row on both sides and half a meter on both the upper and lower plot ends.

Plant traits and parameters tested for summer moong were plant height (cm), DMA (g/plant), LAI, pods per plant, seeds per pod, 1000-seed weight (grams), grain yield (t/ha), consumptive use (mm), crop water productivity (kg/m^3^), irrigation water productivity (kg/m^3^). The plant height was recorded from the ground level to the junction of the leaf’s petiole and the stem of the last fully opened leaf. For DMA, the five plants were selected randomly. They were taken out of the plots and sun-dried, then dried in an oven at a temperature range of 60 °C until the plant sample’s weight remained consistent. The pods were counted at the time of crop harvest, and the results were expressed in the number of pods per plant. For calculating the seeds per pod, the seeds were extracted from the five mature and effective pods manually and then counted. The average value of the seeds was expressed as the number of seeds per pod. The 1000 seeds were selected randomly and weighed with the help of digital top pan balance. The measured value was expressed as 1000 seed weight in grams (g). The grain yield and net plot area of 18.9 m^2^ were recorded from each plot. Then, threshing and cleaning were carried out, and the grain yield was expressed as t/ha.

### Consumptive use of water

The soil water content was measured by gravimetric method from 0 to 10 cm, 10–20 cm, 20–30 cm, 30–40 cm, 40–60 cm, and 60–100 soil profiles, before and after every irrigation. The crop seasonal water uses, or wasteful use of water, was calculated by the formula provided by Singh et al. [33]:

1 $$\begin{gathered}\:C{U_j} = 0.6\:or\:0.8\sum\limits_{k = 1}^N {Ek} \hfill \\\,\,\,\,\,\,\,\,\,\,\, + \sum\limits_{i = 1}^n {\frac{{{M_i} - {M_{ii}}}}{{100}} \times \:{A_{si}} \times \:{D_i} + {E_{RF}}} \hfill \\ \end{gathered}$$

2 $$\:CU = \sum\nolimits_{j = 1}^n {C{U_j}}$$

here, $\:CU$ is the consumptive use of water (cm), and $\sum\nolimits_{j = 1}^n {C{U_j}}$ represents the total use of water (cm) over $\:n$ crop growth intervals, with $\:j$ indicating each individual growth interval, $\:\sum\nolimits_{k = 1}^N {Ek}$ denotes the total actual open pan evaporation across $\:N$ irrigation days, which includes the periods between sampling before and after the $\:{k}^{th}$ irrigation, $\sum\nolimits_{i = 1}^n$ is the cumulative consumptive use of water across $\:n$ intervals between two irrigations, with $\:i$ referring to a single layer within an interval, $\:{M}_{i}$ represents the moisture percentage on a dry weight basis after irrigation in the $\:{i}^{th}$ layer of the soil profile, $\:{M}_{ii}$ denotes the moisture percentage on a dry weight basis before irrigation in the $\:{i}^{th}$ layer of the soil profile, $\:{A}_{si}$ stands for the apparent specific gravity of the $\:{i}^{th}$ layer in the soil profile, $\:{D}_{i}$ indicates the depth of the $\:{i}^{th}$layer of soil (cm), $\:{E}_{RF}$ refers to the effective rainfall (cm) during the $\:{j}^{th}$ growth interval, 0.6 is a constant used for the cooler months (November-February), 0.7 is a constant applied for the months of March-April and September-October, and 0.8 is a constant for the hotter months (May-Aug). To determine the soil profile water contribution, soil water samples were collected from up to 100 cm depth both at crop sowing and at harvest. During the early growth phase, soil water was measured up to 40 cm based on root depth, while in the later stages, soil moisture was recorded from a depth of 0–100 cm.

### Crop and irrigation water productivity

Crop and irrigation water productivity was calculated as [34, 35]:

3 $$\:CWP=\frac{Y}{CU\times\:10}$$

4 $$\:IWP=\frac{Y}{I}$$

where, $\:CWP$ = crop water productivity (kg m^−3^), $\:IWP$ = irrigation water productivity (kg m^− 3^), $\:Y$ = crop yield (kg ha^−1^), $\:CU$ = consumptive use of water (mm), and $\:I$ = irrigation water applied (m^3^).

### Statistical analysis

ANOVA was used to assess the impact of residue management and irrigation regimes on the performance of summer moong. The growth and yield data were analyzed statistically using a split-plot design with SAS software [36], version 9.4 (SAS Institute, Inc., Cary, NC, USA). Mean comparisons were performed using Duncan’s Multiple Range Test at a 5% significance level.

## Results

### Growth parameters

The crop analysis was done based on the various growth parameters, viz., plant height, DMA, and LAI, which varied significantly due to residue management practices and irrigation regimes during both years (Table 4). Residue retention (T_2_) resulted in a 14.6% increase in plant height, an 11.0% increase in DMA, and an 11.5% increase in LAI during 2020; residue retention resulted in a 9.4% increase in plant height, an 8.6% increase in DMA, and 10.3% increase in LAI during 2021 in comparison with straw removal (T_1_). Whereas residue incorporation (T_3_) resulted in a 20% & 15.5% increase in plant height, a 20.8% & 14.0% increase in DMA, and a 26.9% & 20.7% increase in LAI during 2020 and 2021, respectively, in comparison with residue removed treatment. Therefore, the residue retention and residue incorporation were at par with each other except in 2020 for DMA and LAI during 2020 and 2021, where there was a significant relationship.

**Table 4:** Effect of various residue management practices and irrigation regimes on plant height, dry matter accumulation (DMA) and leaf area index (LAI) of the summer mung bean

Treatments	Plant height (cm)					DMA (g plant^− 1^)			LAI		
Residue management	2020	2021		Pooled		2020	2021	Pooled	2020	2021	Pooled
T_1_	40.5b	43.9b		42.2b		17.3c	18.6b	17.9b	2.6c	2.9c	2.7c
T_2_	46.4a	48.0b		47.2a		19.2b	20.2a	19.7a	2.9b	3.2b	3.0b
T_3_	48.6a	50.7a		49.7a		20.9a	21.2a	21.1a	3.3a	3.5a	3.4a
LSD (p = 0.05)	2.8	3.0		1.7		1.4	1.5	0.86	0.2	0.2	0.13
Irrigation scheduling											
I_1_	39.1b	43.5b		41.3b		15.9b	17.6b	16.7b	2.5c	2.8c	2.6c
I_2_	47.5a	48.6a		48.1a		20.6a	20.9a	20.7a	2.9b	3.2b	3.1b
I_3_	48.9a	50.5a		49.7a		21.0a	21.6a	21.3a	3.3a	3.6a	3.4a
LSD (p = 0.05)	2.8	2.9		1.9		0.8	0.9	0.55	0.2	0.3	0.17

The irrigation treatments also showed a significant variation in the various growth parameters of the crop during both years. The I_3_ treatment, in which irrigations were applied at four different growth stages, recorded significantly higher plant height, DMA, and LAI in comparison to I_1_ treatment, in which irrigation was applied at two different growth stages and at par with I_2_ in which irrigation was applied at three different growth stages except for LAI during both years where residue retention and incorporation both were significantly different. The percent increase in plant height, DMA, and LAI was 25.1%, 32.1%, and 32% during 2020, whereas 16.1%, 22.7%, and 28.6% during 2021, respectively, under I_3_ in comparison to I_1_ treatment. Similarly, the percent increase in plant height, DMA, and LAI was 21.5%, 29.6%, and 16% during 2020, and it was 11.7%, 18.7%, and 14.3%, respectively, under I_2_ in comparison to I_1_ during 2021.

Pooled analysis of the growth parameters (Table 4) stated that the plant height, DMA and LAI values were significant in terms of variation over the year at a 5% level of significance. There was 4.5% improvement in DMA and 8.9% in LAI during 2021 over 2020. The effect of residue was significant in a pooled analysis of the plant height, DMA, and LAI. The interactive effect of year with residue, year with irrigation, residue with irrigation, and residue along with irrigation and year was non-significant except for the impact of year x irrigation on the DMA. Residue incorporation (T_3_) and residue retention (T_2_) were at par with each other for plant height and DMA and significantly different from non-residual (T_1_) plots. There was a significant difference in LAI between T_3_ and T_2_; both significantly differed from T_1_. There was a 17.7%, 17.3%, and 23.4% improvement in plant height, DMA, and LAI under T_3_ and an 11.8%, 9.7%, and 10.5% increase under (T_2_) compared to T_1_. T_3_ also showed an 11.7% increase in LAI over T_2_.

The effect of irrigation (Table 4) showed a significant variation in plant height, DMA, and LAI based on a pooled data comparison. Irrigation applied at four different growth stages (I_3_) showed a significant improvement in plant height, DMA, and LAI over the irrigation applied at two different growth stages (I_1_). The treatment where two irrigations were applied (I_2_) was at par with I_3_ except for LAI, where they had a significant difference. The I_3_ resulted in 20.2%, 27.4%, and 28.4%, whereas I_2_ resulted in 16.4%, 24.2%, and 15.7% increase in plant height, DMA, and LAI over I_1_. I_3_ showed a 10.8% significant increase in LAI over I_2_.

Results in Table 4 showed that the DMA under I_1_ was significantly lower than I_2_ and I_3_ during both years. A comparison of different years at each level of irrigation showed that DMA during 2020 under I_1_ was considerably lower than all other treatments and other treatments were at par with each other.

### Yield parameters

The application of wheat residue, either as retention or incorporation, significantly affected the various yield-attributing parameters: pods per plant, seeds per pod, and 1000-seed weight (Table 5). The maximum value of pods per plant was found under T_3_ treatment (24.3 plant^-1^), which was significantly different from T_1_ (21.6 plant^-1^) and did not differ from T_2_ (23.5 plant^-1^) during 2020. Similar results were also obtained in 2021 for T_3_ (25.8 plant^-1^), T_2_ (24.5 plant^-1^) and T_1_ (21.9 plant^-1^), respectively. There was a 12.5% increase in T_3_ and an 8.8% increase in T_2_ compared to T_1_ during 2020. Similarly, in 2021, there was a 17.8% increase in T_3_ and an 11.9% increase in T_2_ compared to T_1_ treatment. Among the irrigation treatments, a significant difference was obtained during both the years 2020 and 2021 between I_3_ (24.5 & 25.5 plant^-1^) and I_1_ (21.3 & 22.2 plant^-1^), as well as between I_1_ and I_2_ (23.6 & 24.5 plant^-1^). The percent reduction of I_2_ in comparison with I_3_ was 3.7%, whereas the percentage reduction in I_1_ was 13.1% during 2020, and during 2021, it was 3.9% in I_2_ and 12.9% in I_1_ compared with I_3_. The effect of interaction among residue management and irrigation regimes on pods per plant was non-significant during both years.

**Table 5:** Effect of residue management practices and irrigation regimes on pods per plant, seeds per pod and 1000-seed weight of summer mung bean

Treatments	Pods per plant				Seeds per pod			1000-seed weight (g)		
Residue management	2020	2021	Pooled		2020	2021	Pooled	2020	2021	Pooled
T_1_	21.6b	21.9b	21.8b		9.2	9.0b	9.1b	44.8c	45.2c	45.0c
T_2_	23.5a	24.5a	24.0a		9.4	9.6ab	9.5ab	46.0b	46.4b	46.2b
T_3_	24.3a	25.8a	25.0a		9.7	9.9a	9.8a	47.3a	47.8a	47.6a
LSD (p = 0.05)	1.6	1.6	0.93		NS	0.6	0.33	0.9	1.0	0.56
Irrigation scheduling										
I_1_	21.3b	22.2b	21.7b		8.9b	8.9b	8.9b	45.1b	45.5b	45.3b
I_2_	23.6a	24.5a	24.1a		9.6a	9.6a	9.6a	46.3a	46.7a	46.5a
I_3_	24.5a	25.5a	25.0a		9.9a	10.0a	9.9a	46.7a	47.2a	47.0a
LSD (p = 0.05)	1.9	2.0	1.30		0.3	0.4	0.26	1.1	1.1	0.73

Pooled analysis (Table 5) of the data containing the number of pods per plant showed a similar trend, and there was a significant result in terms of year, effect of residue, and effect of irrigation, whereas other interaction effects were non-significant. There was a 4% significant improvement in 2021 for the number of pods per plant over 2020. The number of pods per plant was at par with residue retention and incorporation, whereas both these treatments had a significant difference with no-residue plots. There was a 10.2% and 15% increment in pods per plant under residue retention (24.0 plant^-1^) and residue incorporation (25.0 plant^-1^) in comparison with residue removal (21.8 plant^-1^). The irrigation treatments I_3_ (25.0 plant^-1^) and I_2_ (24.1 plant^-1^) showed a significant increment by 15.2% and 11.1% over I_1_ (21.7 plant^-1^) treatment, whereas I_3_ and I_2_ were at par with each other.

Similarly, T_3_ recorded the highest number of seeds per pod (Table 5) during the year 2021 (9.9 pods^-1^), which was significantly higher than T_1_ (9.0 pod^-1^) and at par with T_2_ (9.6 pod^-1^), respectively. In contrast, the effect of residue was non-significant during the year 2020. T_2_ was also at par with T_1_ in 2021. Among the irrigation regimes, the I_3_ treatment (9.9 & 10.0 pod^-1^) was significantly more than the I_1_ treatment (8.9 pod^-1^) and at par with I_2_ (9.6 & 9.6 pod^-1^) during both years. The influence of the interaction between residue management and irrigation systems on the number of seeds per pod was found to be insignificant for both years.

A pooled analysis (Table 5) conducted over both the years for the number of seeds per pod revealed no substantial changes. In contrast, the residue and irrigation effects were significant, and the remaining interactions were non-significant. There was a significant difference only between residue incorporated and residue removed treatment. There was an 8.2% significant improvement in the number of seeds per pod with wheat residue incorporation over residue removal. Scheduling irrigation at four different growth stages (I_3_) and three different growth stages (I_2_) showed a significant improvement in the number of seeds per pod compared with scheduling at two different growth stages (I_1_). I_3_ (9.9 pods^− 1^) recorded 11.2% and I_2_ (9.6 pods^− 1^) recorded 7.7% increase in seeds per pod over I_1_ (8.9 pods^− 1^).

The trend was also similar for 1000-seed weight (Table 5); the maximum value was obtained under the T_3_, significantly different from the T_2_ and T_1_. The percent reduction in weight was 2.7% in T_2_, whereas in T_1_, it was 5.3% in comparison with T_3_ during 2020, and there was a 2.9% reduction in T_2_ and a 5.4% reduction in T_1_ compared with T_3_ in the year 2021. The treatment I_3_ was reported to have a significantly higher 1000-grain weight than the I_1_ and at par with I_2_ during both years. There was a 2.6% and 3.4% reduction in I_1_ compared with I_2_ and I_3_ during 2020 and a 2.6% and 3.6% reduction in I_1_ compared to I_2_ and I_3_ during 2021.

The pooled data on 1000-seed weight resulted in a significant effect for the year, residue, and irrigation, along with the interaction of residue and irrigation. There was a considerable improvement in 1000-seed weight in the year 2021 over the year 2020. Residue-incorporated and retained treatment showed a significant 5.7% and 2.6% increment in 1000-seed weight compared to no residue treatment. Residue incorporation also showed a 3% improvement over residue retention. The irrigation treatment containing four (I_3_) and three (I_2_) irrigations were at par with each other, significantly different from two (I_1_) irrigations. There was a 3.7% and 2.6% improvement in I_3_ and I_2_ over I_1_. The various levels of residues at each level of irrigation (Table 5) shows that when there were only two irrigations (I_1_) the residue incorporation gave a significantly higher 1000-seed weight than removal. However, residue retention was at par with both residue incorporation and residue removal. The residue effect was non-significant in the treatment where three irrigations were applied. In the treatment where four irrigations (I_3_) were used, the residue incorporation gave significantly higher 1000-seed weight than residue retention and no residual treatment. The residue removed and residue retention treatment did not differ.

### Grain yield

The most critical factor for evaluating and contrasting the efficacy of experimental variables is grain yield. It depends on various characteristics, including the number of pods per plant, the number of seeds per pod, and the weight of a thousand seeds. Table 6 provides information on how residue management practices and irrigation planning affect the yield of mung bean grains. The results revealed that among three different residue management practices, the maximum grain yield was observed under residue-incorporated plots T_3_ (0.69 t ha^-1^ during 2020 and 0.74 t ha^-1^ during 2021), and the lowest was observed under treatment where residue was removed from the field (T_1_) during both years. The residue incorporated T_3_ was significantly higher than T_1_, where residue was removed from the field, whereas it was at par with T_2_ (0.65 t ha^-1^ and 0.71 t ha^-1^ during 2021). The percent increase in grain yield was 18.2% and 25.5% during 2020, and it was 16.4% and 21.3%, respectively, during 2021 under T_2_ and T_3_ in comparison to T_1_ treatment.

**Table 6:** Interaction effect of residue management practices and irrigation regimes on the grain yield of summer mung bean

Treatments	Grain yield (t/ha)		
Residue management	2020	2021	Pooled
T_1_	0.55b	0.61b	0.58b
T_2_	0.65a	0.71a	0.69a
T_3_	0.69a	0.74a	0.72a
LSD (p = 0.05)	0.07	0.07	0.04
Irrigation scheduling			
I_1_	0.48b	0.56b	0.52b
I_2_	0.69a	0.72a	0.71a
I_3_	0.70a	0.79a	0.76a
LSD (p = 0.05)	0.08	0.06	0.05

**Fig. 3:**
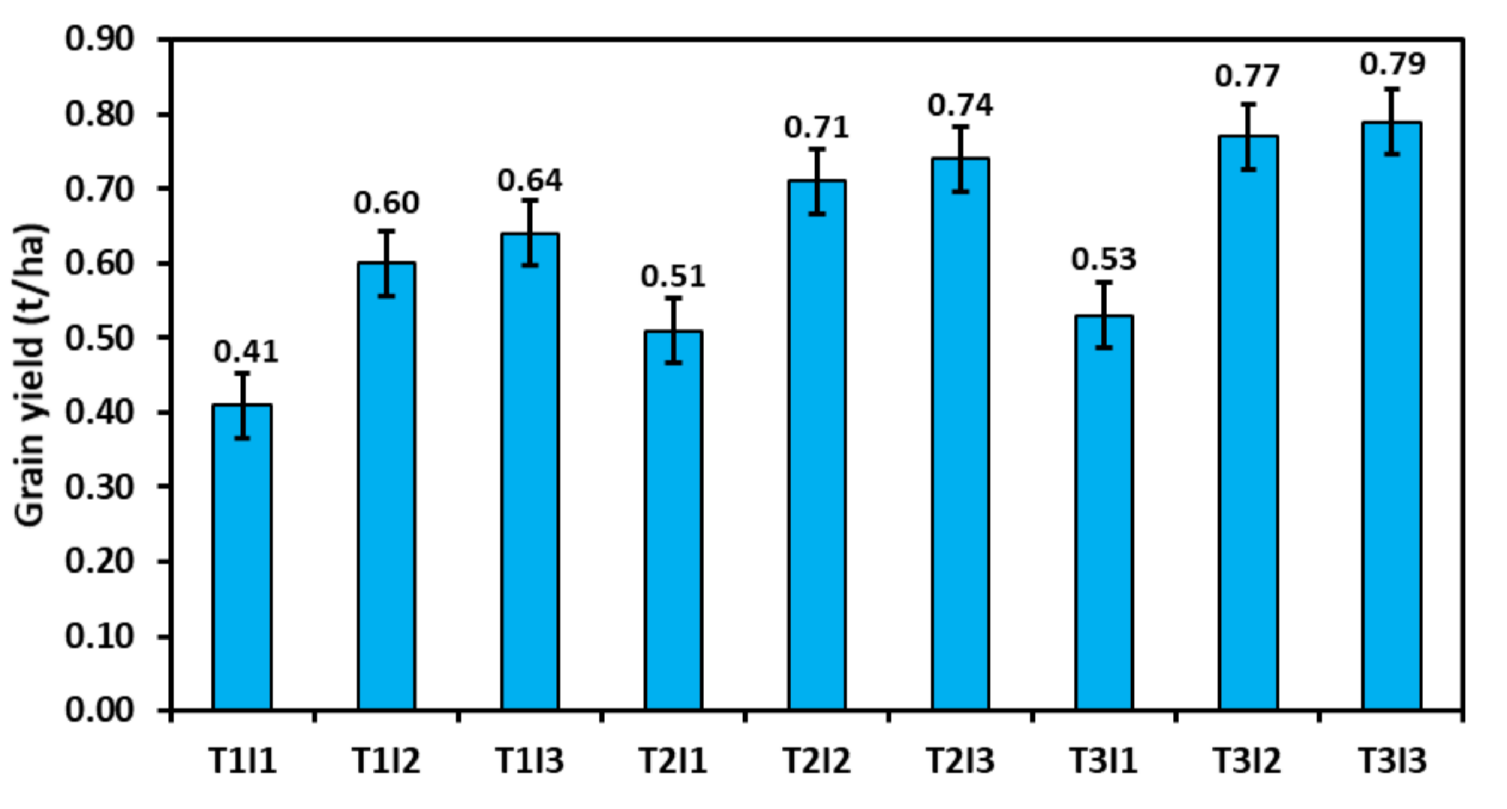
Effect of various residue management practices and irrigation schedules on grain yield of summer mung bean during 2020

**Fig. 4:**
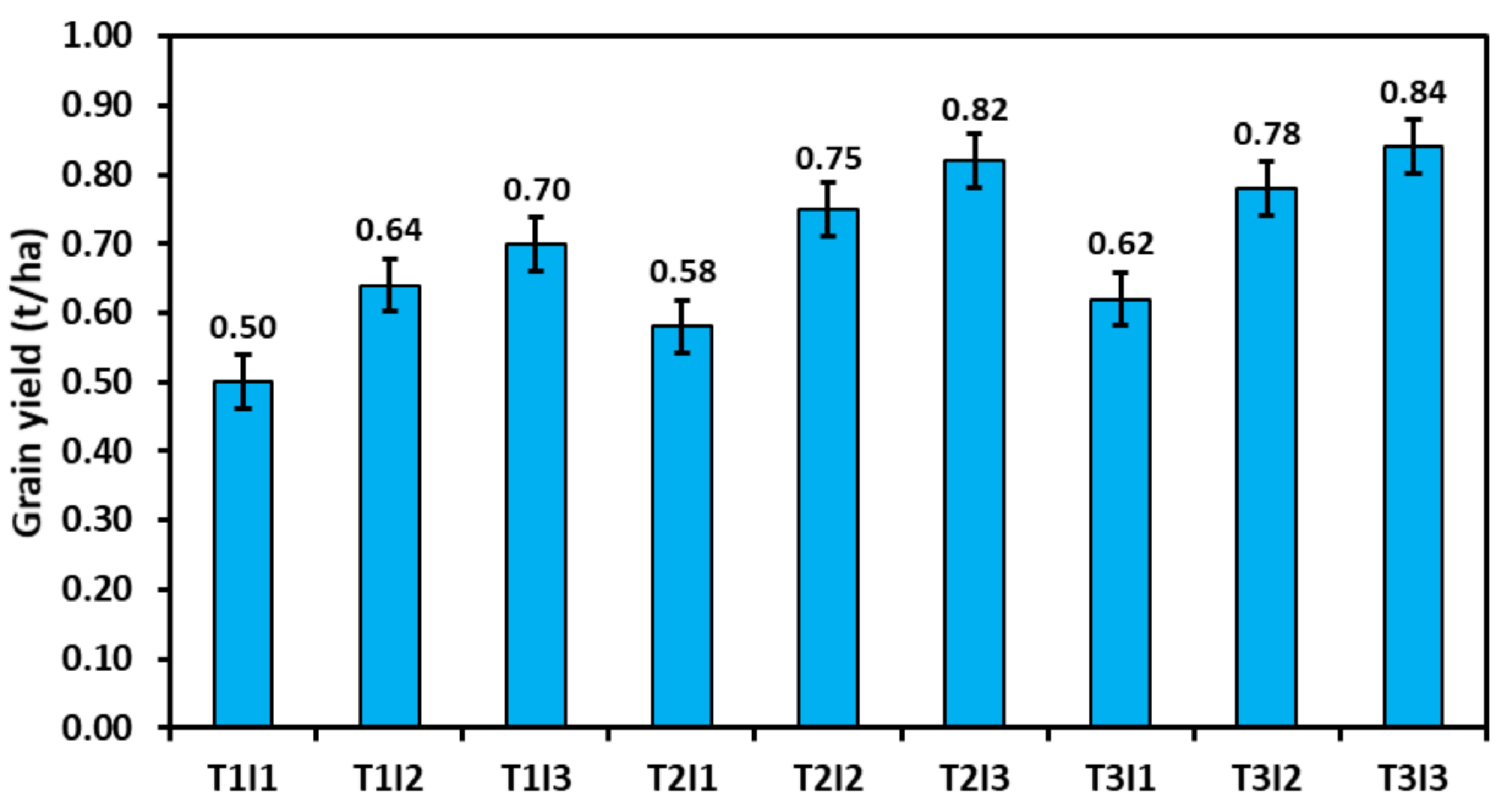
Effect of various residue management practices and irrigation schedules on grain yield of summer mung bean during 2021

The irrigation treatment (Table 6), too, showed a significant effect as the maximum yield of the crop was found to be under I_3_ treatment (0.70 t ha^-1^ in 2020 and 0.79 t ha^-1^ in 2021). In contrast, the lowest yield was observed under the I_1_, i.e., when irrigation was applied at two different crop growth stages compared to four growth stages during both years. The yield obtained in I_3_ was significantly higher than I_1_, whereas it was at par with I_2_. The percent increase in yield obtained was 45.8% and 43.8% during 2020, whereas it was 41.1% and 28.6% during 2021 under I_3_ and I_2_, respectively, compared to the I_1_ treatment. In treatment combination, T_1_I_3_ produced 0.64 t ha^-1^ seed yield whereas T_2_I_2_ and T_3_I_2_ gave 0.71 and 0.77 t ha^-1^ of seed yield under three irrigations (Fig. 3), which were more outstanding than grain yield gained under four irrigations. A similar trend was there in 2021 (Fig. 4), and treatment T_2_I_2_ and T_3_I_3_ receiving four irrigations resulted in 0.75 and 0.78 t ha^-1^ seed yields, higher than T_1_I_3_ (0.70 t ha^-1^) receiving four irrigations.

Pooled analysis of the yield shows a significant effect of year, residue, and irrigation regimes, and other interaction effects were non-significant. There was a 9.3% increase in the yield of summer mung bean during 2021 over the year 2020. Adding wheat residue to summer mung bean resulted in a significant increment in the yield of summer mung bean. At the same time, there was no significant difference between residue retention (0.69 t ha^− 1^) and residue incorporation (0.72 t ha^− 1^). Residue incorporation increases yield by 24.1%, whereas residue retention resulted in a 18.9% yield improvement over the residue removed treatment (0.58 t ha^− 1^). The irrigation under I_2_ (0.71 t ha^− 1^) and I_3_ (0.76 t ha^− 1^) resulted in significant yield improvement over-irrigation scheduled under I_1_ (0.52 t ha^− 1^). There was no significant difference between I_2_ and I_3_; thus, one extra irrigation can be avoided to gain the same yield and conserve the crop’s water use.

### Consumptive use

The consumptive use (CU, mm) or crop water use under various residue management practices and irrigation regimes is given in Table 7. The maximum crop water use was obtained under T_1_ (299.9 mm), and the lowest was observed under T_2_ (290.8), which was followed by T_3_ (291.3 mm) during 2020. T_2_ recorded 9.1 mm lower water use than T_1_. However, during 2021, the maximum value of consumptive use was observed under the T_2_ (312.9 mm) treatment, whereas the lowest consumptive use was obtained under the T_1_ (292.2 mm) treatment and was followed by T_3_ (301.2 mm). The consumptive use was 11.7 mm lower under T_3_ and 20.7 mm lower under T_1_ than T_2_ (312.9 mm). Applying residue as incorporation has helped reduce the crop’s water use. Pooled analysis showed the highest CU of water under T_2_ (301.8 mm), followed by T_3_ (296.3 mm) and T_3_ (296.1 mm).

**Table 7:** Effect of residue management practices on the consumptive use (CU), crop water productivity (CWP) and irrigation water productivity (IWP) of summer mung bean

Treatments	Consumptive use (mm)			Crop water productivity (kg m^− 3^)			Irrigation water productivity (kg m^− 3^)		
	2020	2021	Pooled	2020	2021	Pooled	2020	2021	Pooled
Residue management									
T_1_	299.9	292.2	296.1	0.18b	0.21b	0.20b	0.25b	0.28b	0.27b
T_2_	290.8	312.9	301.8	0.23a	0.23ab	0.23ab	0.30a	0.33ab	0.32ab
T_3_	291.3	301.2	296.2	0.24a	0.25a	0.24a	0.32a	0.34a	0.33a
LSD (p = 0.05)	-	-	-	0.03	NS	0.02	0.05	NS	0.02
Irrigation scheduling									
I_1_	225.7	253.5	239.6	0.21ab	0.22a	0.22a	0.32a	0.38a	0.35a
I_2_	292.3	312.5	302.4	0.24a	0.23a	0.23a	0.31a	0.32b	0.31b
I_3_	364.1	340.3	352.1	0.20b	0.23a	0.22a	0.24b	0.26c	0.25c
LSD	-	-	-	0.03	NS	NS	0.04	0.05	0.02

The I_3_ (364.1 mm) treatment of the irrigation regime (Table 7) has been found to increase CU of the crop in comparison with I_2_ (292.3 mm) and I_1_ (225.7 mm) during 2020, and a similar trend was in 2021 as maximum water use was in I_3_ (340.3 mm) followed by I_2_ (312.5 mm) and I_1_ (253.5 mm). During 2020, I_3_ used 71.8 mm more water than I_2_, and 138.4 mm of water was added to I_1_. Similarly, in 2021, I_3_ used 27.8 mm and 86.8 mm additional water than I_2_ and I_1_ treatments. Similar results were also obtained under pooled analysis conditions, and I_3_ (352.1 mm) recorded the highest CU, followed by I_2_ (302.4 mm) and I_1_ (239.6 mm).

### Crop water productivity and irrigation water productivity

Table 7 presents the data that illustrates the impact of different residue management methods and irrigation systems on the productivity of the summer mung bean crop and its irrigation water productivity (IWP). The maximum crop water productivity (CWP) was observed under the residue-incorporated treatment T_3_ (0.24 & 0.25 kg m^-3^), which was followed by residue-retained treatment T_2_ (0.23 & 0.23 kg m^-3^), and the least amount was observed under residue removal treatment T_1_ (0.18 & 0.21 kg m^-3^) during 2020 and 2021 respectively. A similar trend was also obtained for IWP. Among the irrigation regimes, the I_2_ treatment gives maximum CWP (0.24 kg m^-3^), which was followed by I_1_ (0.21 kg m^-3^) and I_3_ (0.20 kg m^-3^) during 2020. Meanwhile, in 2021, the I_2_ and I_3_ treatments (0.23 kg m^-3^) gave the same CWP as the I_1_ treatments (0.22 kg m^-3^). However, there was a different trend for IWP as the highest IWP was observed under the I_1_, which I_2_ and I_3_ followed. The IWP under I_1_, I_2_, and I_3_ were 0.32, 0.31, and 0.24 kg m^-3^ during 2020 and 0.38, 0.32 and 0.26 kg m^-3^, respectively, during 2021. The pooled analysis stated a similar trend where the CWP was maximum under T_3_ (0.24 kg m^-3^) followed by T_2_ (0.23 kg m^-3^) and T_1_ (0.20 kg m^-3^). The IWP also followed a similar trend and maximum values were obtained under T_3_ (0.33 kg m^-3^), T_2_ (0.32 kg m^-3^), and T_1_ (0.27 kg m^-3^). Among irrigation water treatments, I_2_ (0.23 kg m^-3^) recorded maximum CWP, followed by I_3_ (0.22 kg m^-3^) and I_1_ (0.22 kg m^-3^) having similar values. The IWP was maximum under I_1_ (0.35 kg m^-3^), followed by I_2_ (0.31 kg m^-3^) and I_3_ (0.25 kg m^-3^).

### Soil properties

**Fig. 5:**
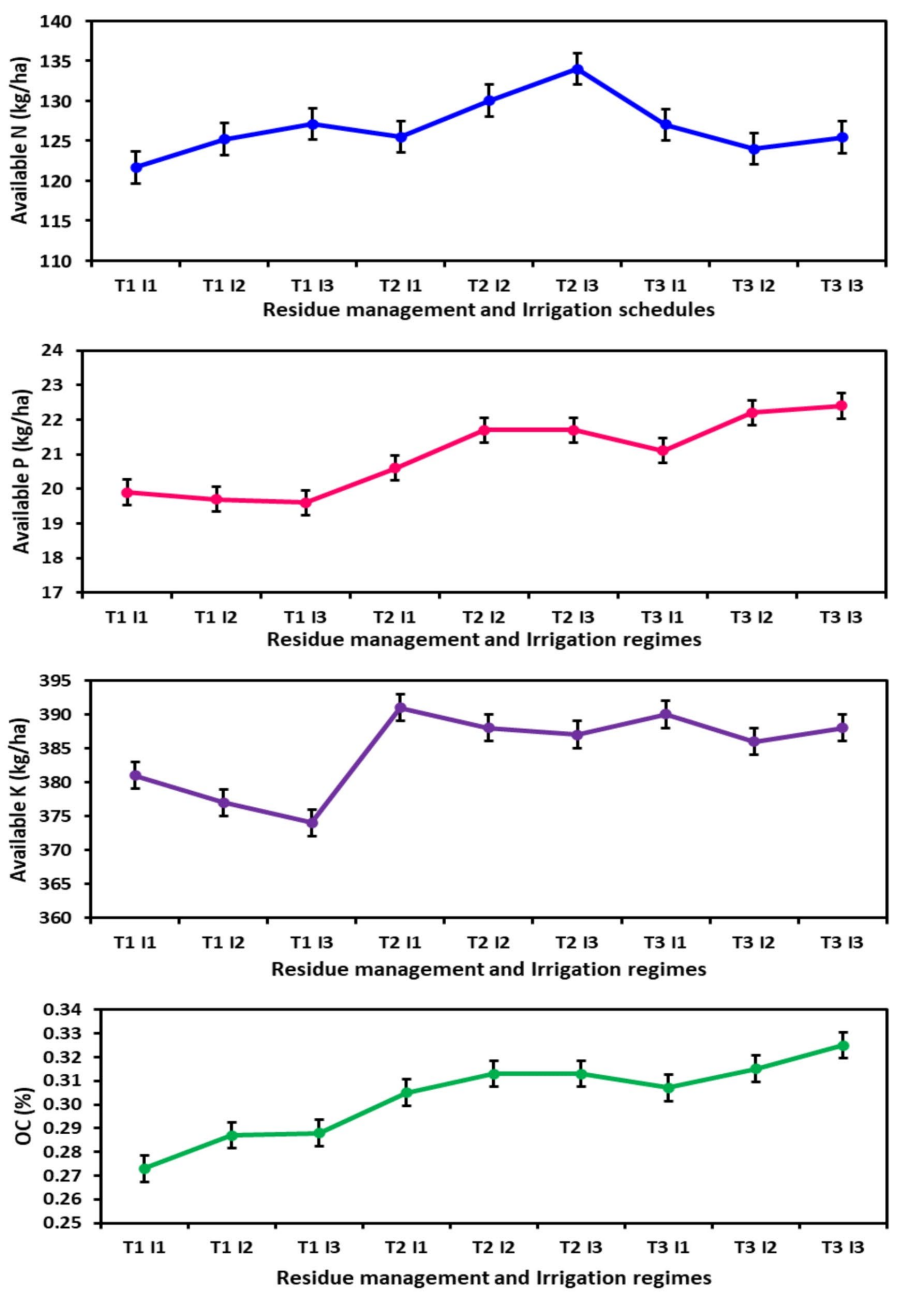
Effect of different wheat residue management practices and irrigation regimes on soil properties at the completion of the experiment

The chemical analysis was done after the experiment and given in Fig. 5. In T_3_I_3_, we observed the peak values for organic carbon (OC) and available Phosphorus (P). This was due to the process of integrating the residual wheat material left after the creation of wheat straw, in conjunction with preparatory tillage. This process was further enhanced by four irrigation stages: the vegetative stage, the flowering stage, the pod formation stage, and the pod filling stage. At the same time, the available nitrogen (N) was highest in fields where leftover wheat residue with zero tillage was done, along with four irrigations at various critical stages (T_2_I_3_). The lowest OC and N were observed in treatment with no wheat residue in the field and conventional tillage with two irrigations (T_1_I_1_). Available potassium (K) was also highest in T_2_ treatment but with two irrigations (T_2_I_1_). The lowest K content was noted in T_1_I_3_, in treatment wherever crop residue was removed and four irrigations were administered. Therefore, both the treatments - incorporating residue and zero tillage with leftover residue, resulted in an increase in the soil’s OC, as well as the available N, P, and K content in the surface soil.

## Discussion

The reception of a pulses-based cropping system has been supported as an option in contrast to the persistent rice-wheat cropping system because of its ability to renew soil fruitfulness by fixing environmental nitrogen. The mid-year mung bean crop fits well in the *kharif* maize-wheat-summer mung bean or rice-wheat-summer mung bean cropping system [37]. After harvesting, the wheat straw has to be managed by removing, incorporating, or sowing the next crop without incorporation or zero tillage. It conserves energy and also saves labor inputs. Thus, zero tillage and residue incorporation affect various growth and yield parameters. Summer mung bean is grown during the season when the crop’s water requirement is at its peak [38], so enhanced irrigation strategies that yield more with lesser irrigation must be the primary concern, along with moisture conservation practices. Thus, the higher increase in plant height could be attributed to moisture conservation in residue-treated plots [39]. Better moisture availability at critical growth stages leads to increased plant height, as Yadav and Singh [40] reported. Meena et al. [41] reported a better N, P, and K uptake in crops applied with the crop residue, leading to increased DMA. Prevention of abiotic stress and high temperature and water stress also leads to increased DMA in moong bean, as Nath et al. [42] reported. Since drought declined the plant pigments and leaf water content, crop growth and yield declined [43–45]. Also, deficit water causes lipid peroxidation and disturbs the balance of osmolytes and nutrients in plants resulting in abnormal growth [46–48]. The drought stress reduces cell turgor, leading to reduced cell expansion, ultimately influencing plant height, increasing cell proline content, and affecting the leaves’ chlorophyll content [49]. The higher LAI obtained under I_3_ was probably due to higher irrigation frequency, leading to better vegetative growth and active cell division. Insufficient irrigation can also have adverse effects on LAI and crop growth. An inadequate water supply can lead to water stress, which can cause leaves to wilt, reduce photosynthesis, and ultimately decrease LAI. One of the initial signs of moderate dehydration is decreased transpiration following stomatal closure. The shoot’s water condition is stabilized by limiting the amount of water lost to the atmosphere [50]. The production, transport, and intercellular and subcellular repartitioning of abscisic acid facilitate stomatal closure during dehydration [51]. The water stress can also reduce the root’s uptake of nutrients and minerals, leading to nutrient deficiency and reduced LAI. The water stress inhibits the enlargement of cells and reduces their optimum development, as also reported by Uddin et al. [52] who also state that root growth is affected by moisture stress. Plant growth substances such as cytokinins, auxins, gibberellins, salicylic acid, and abscisic acid modulate plant responses toward drought [53]. As I_3_ out-yielded remaining irrigation treatments in terms of LAI, the reason could be due to better vegetative growth under assured irrigation supply and proper sugar accumulation inside the leaves, which resulted in optimum LAI of the crop [54].

The number of pods per plant, seeds per pod, and 1000-seed weight are essential traits as they estimate the grain yield per unit area, which is helpful for crop management and planning. Our study reported an increase in the number of pods per plant with the addition of residue, either as residue retention or residue incorporation as plant development was better due to better utilization of the resources [55]. The number of seeds per pod reduced significantly when stress occurs at the reproductive stage; however, Simsek et al. [56] reported a more pronounced impact of water stress at the vegetative stage than the reproductive stage. The number of seeds per pod was considerably better in residue-retained and residue-incorporated plots. Improved tillage practices, such as conservation agriculture, permanent bed cover, and residue retention practices significantly increased the yield attributes in chickpeas, such as pods per plant and 1000-seed weight [57].

The augmentation in the accumulation of dry matter, resulting from a higher frequency of irrigation, contributes to a notable increase in the number of pods per plant. Asaduzzaman et al. [58] reported similar findings. Moreover, critical irrigation scheduling at pod formation and filling stages leads to better pod retention. Irrigation also had a positive impact on the number of seeds per pod, leading to an increase. This is because the absence of irrigation during the pod formation and development stages influenced seed production. The possible reason could be that the crop maturity was delayed by increased irrigation frequency, which gave the photosynthates more time to partition and assimilate into the seeds. Omid [59] also reported similar results. In our study, I_1_ recorded the lowest 1000-seed weight and the maximum being observed under I_3_, which shows the negative impact of skipping irrigation at the pod formation or filling stage. The plant’s developmental stage, the severity and length of the stress, the genotypic capability of the species, and environmental interactions all have a role in the impact of drought stress. This result was per Sadaf and Tahir [60].

Applying crop residue mulch in the green gram crop can help increase crop yield and crop water productivity [61]. The grain yield of the crop was reduced significantly when the crop was under stress at the pod formation stage, which was also reported by Islam et al. [18]. Adding crop residue improved soil properties such as aggregate stability, infiltration, soil moisture conservation, and soil organic matter, resulting in increased crop yield [62]. Similarly, our results revealed that among three different residue management practices, the maximum amount of grain yield was observed under residue-incorporated plots T_3_, and the lowest was observed under treatment where residue was not incorporated T_1_ during both years. The grain yield in T_3_ was significantly higher than in T_1_, where residue was not incorporated, whereas it was at par with T_2_. Thus, applying residue as a surface application or incorporation increases the crop yield. Mohammad et al. [63] also reported increased crop yield and water use efficiency due to zero tillage and residue retention practices. The increasing yield due to residue incorporation has also been reported by Suryavanshi et al. [64], and this could help compensate for the yield loss due to limited water conditions.

**Fig. 6:**
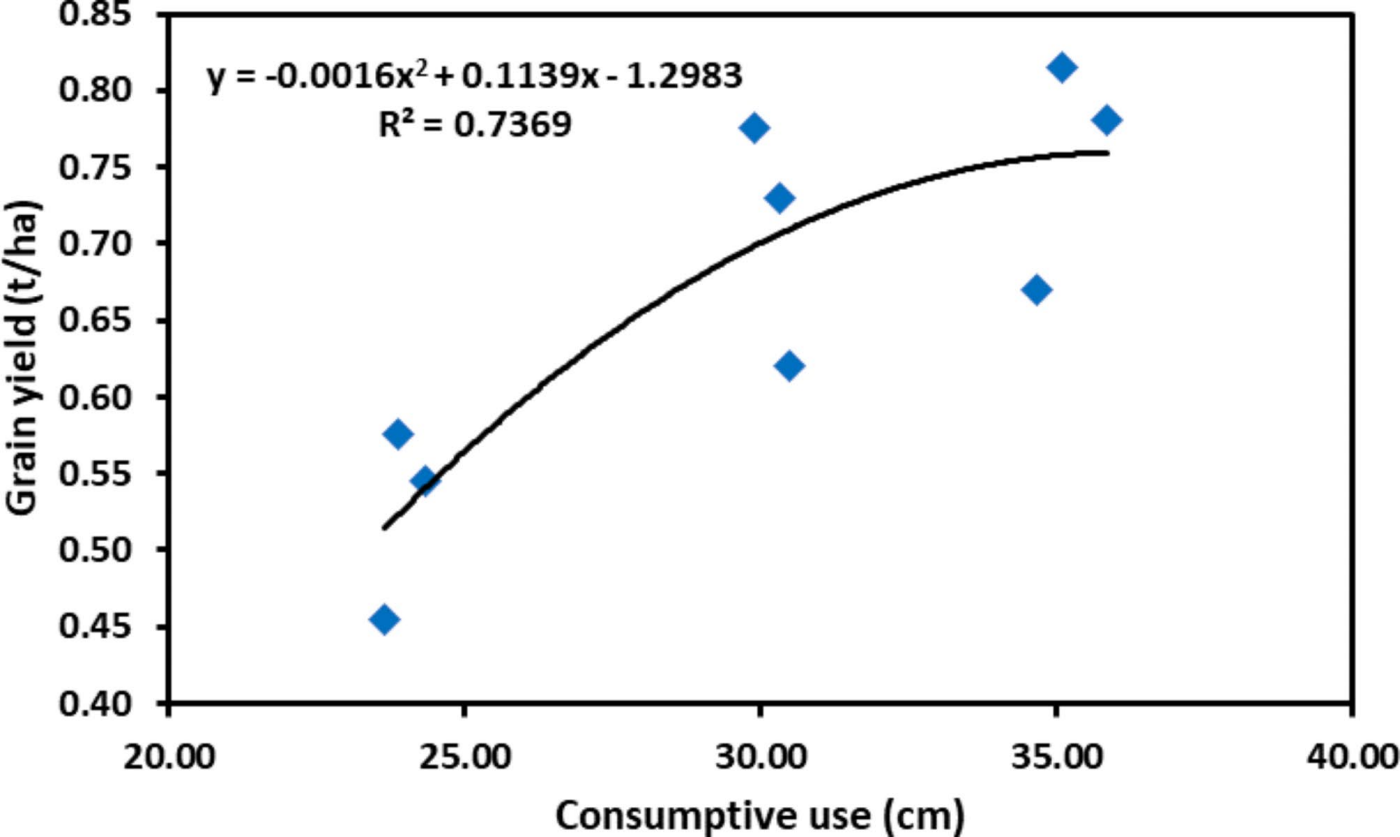
Relationship between grain yield and total consumptive use under various treatments

Water application at the early flowering and early pod formation stage increases the green gram’s grain and straw yield compared to rainfed cultivation [65]. Lesser irrigation applications to crops lead to a significant reduction in the yield, and the effect is more pronounced when water stress occurs at the reproductive stage [66, 67] as summer mung bean is grown during the season when the crop’s water requirement is at its peak [38]. The maximum yield of the crop was found to be under the I_3_ treatment. In contrast, the lowest yield was observed under the I_1_, i.e., when irrigation was applied at two different crop growth stages compared to four different growth stages. The yield obtained in I_3_ was significantly higher than I_1_, whereas it was at par with I_2_, thus indicating the importance of scheduling irrigation at the pod formation or pod filling stage. Mandai et al. [68] also documented that the grain yield of the crop improved when irrigation was applied during the vegetative, flowering, and pod development stages. The higher grain yield of I_3_ and I_2_ compared to I_1_ treatments resulted from the higher pod set, a more significant number of seeds per pod, and higher 1000-grain weight, all influenced by the higher moisture availability at the I_2_ and I_3_. In our study, the combination of T_2_I_2_ and T_3_I_2_ yields more than T_1_I_3_, where one extra irrigation was applied during both years (Figs. 3 and 4). In this study, it is perceived that the mung bean yield is nearly related to consumptive use (*r* = 0.74), evident in Fig. 6.

The maximum CWP was observed under treatment T_3_, which treatment T_2_ followed, and the least amount of value was observed under treatment T_1_ during both years. The low consumptive use under the residue-incorporated treatments and significantly higher yield observed under the same plot give higher crop water productivity. Among the irrigation regimes, the I_2_ and I_3_ treatments result in higher crop water productivity than I_1_. The lower yield was accompanied by lesser water use in I_1_. In contrast, the high yield was accompanied by high water use in I_2_ and I_3_, thus giving almost similar amounts of crop water productivity during 2021. In contrast, in 2020, I_2_ recorded higher CWP than other treatments. Kaur et al. [69] also reported that applying a smaller amount of water can achieve a similar yield level per unit of irrigation water. The IWP was highest under T_3_ due to the maximum yield produced compared to other residue management practices. Adding residue increased IWP due to increased crop yield [42]. Among the irrigation schedules, the I_1_ recorded the highest IWP because the minimum amount of irrigation water was supplied under this (150 mm), and the highest was applied under the I_3_ (300 mm). The study also reported increased wasteful use of the crop with increasing frequency. Applying crop residue in the permanent bed system under green gram improved crop water productivity [70]. The I_1_ treatment recorded maximum IWP, and I_3_ stated that deficit irrigation increased IWP [71]. This was because the minimum amount of irrigation (150 mm) was applied under I_1_ compared to I_3_, where 300 mm of water was used. The yield increase could not overcome the efficiency of irrigation-applied water. However, irrigation application at some critical stages can be avoided, and a comparable yield can be obtained with less irrigation frequency.

The treatments of incorporating residue and zero tillage with leftover residue both led to an increase in the soil’s OC, as well as the available N, P, and K content in the surface soil. Thus, lowermost OC and N, P K were observed in treatment with no wheat residue in the field and conventional tillage. Ghuman and Sur [72] also reported higher OC under minimum tillage and crop residue mulch. The increased OC under minimum tillage may be due to lesser oxidation of in situ organic matter owing to the absence of tillage [73, 74] and absenteeism of soil redistribution. Tripathi et al. [75] and Rashid et al. [76] reported increased soil organic carbon due to cereal residue incorporation. A similar study also reported the potential benefit of no-tillage in conserving water under semi-arid conditions. Reduced soil erosion, surface runoff, organic matter mineralization, and organic carbon content are typically better in soils where conservation tillage has been practiced than conventional tillage [77]. It was further evaluated that OC, N, and P content were higher under a more significant number of irrigations. The higher moisture level in the soil helped the availability of various nutrients. That number of irrigations increased the different chemical properties of soil (OC, N, and K) while K content was maximum, where fewer irrigations were given to crops. The K availability may be higher under lesser moisture content in the soil.

## Conclusion

Wheat residue addition is beneficial in increasing summer mung bean productivity and crop water productivity. The residue incorporation can increase the grain yield by an average of 24.1%, and residue retention increases by 19.0% compared to residue removal. Moreover, applying crop residue can help minimize water use and increase crop productivity. For example, one extra irrigation at the pod-filling stage under summer moong increased the grain yield by 36.5%, and one additional at the pod-formation stage increased by 46.2% compared to two irrigations at the vegetative and flowering stages. Therefore, the wheat residue incorporation in summer mung bean can help save one irrigation.

In addition to the immediate benefits observed in terms of increased productivity and water savings, the adoption of wheat residue incorporation in summer mung bean cultivation holds broader implications for sustainable agriculture and environmental stewardship. By harnessing the potential of crop residue as a soil amendment, farmers can promote soil health and fertility, leading to improved long-term productivity and resilience of cropping systems.

The findings from the presented study highlight the multifaceted benefits of incorporating wheat residue in summer mung bean cultivation, ranging from immediate yield improvements and water savings to long-term soil health and environmental sustainability. By promoting the adoption of residue management practices and efficient irrigation strategies, agricultural stakeholders can contribute to the development of resilient and resource-efficient cropping systems that meet the dual objectives of increasing agricultural productivity and conserving natural resources for future generations.

## Data Availability

All the data & materials related to this paper will be available on request by the corresponding author.
